# Micro Fracture Behavior of Composite Honeycomb Sandwich Structure

**DOI:** 10.3390/ma14010135

**Published:** 2020-12-30

**Authors:** Guangjian Bi, Jianping Yin, Zhijun Wang, Zijian Jia

**Affiliations:** 1 College of Mechanical and Electrical Engineering, North University of China, Taiyuan 030051, China; bgjnuc@163.com (G.B.); wzj@nuc.edu.cn (Z.W.); 2 Science and Technology on Transient Impact Laboratory, P.O. Box, Beijing 102202, China; zjjr116@163.com

**Keywords:** composite honeycomb structure, fracture energy, energy absorption rate, numerical simulation

## Abstract

To study the influence of structure size and composite forms on the mechanical properties of the composite double honeycomb sandwich structure, a composite double honeycomb sandwich structure was initially designed. The dynamic response of a composite double-layer honeycomb sandwich structure under high-speed impact was studied through theoretical analysis and numerical simulation. Ls-dyna software was used to simulate the initially designed composite structure. According to the numerical simulation results and the proposed method for calculating the fracture energy of the composite double honeycomb sandwich structure, the effects of different composite forms on the mechanical properties were analyzed. The results show that the proposed fracture energy calculation method can effectively describe the variation trend of the honeycomb structure and the micro-element fracture situation in the valid time. The fracture energy curve has a high sensitivity to cell density and material, and the strength of the top core has a great influence on the overall energy absorption. Compared with the traditional honeycomb protection structure, the energy absorption of the initially designed composite honeycomb sandwich structure was improved effectively.

## 1. Introduction

Rigid sandwich structures are widely used in military fields such as aerospace, shipping, and armored vehicles because of their high specific strength, specific rigidity, specific energy absorption, and other characteristics. With the continuous advancement of sandwich structure research, there has been much work carried out on the optimal design of the sandwich structure in order to meet the acoustics, thermal, and mechanical property requirements for different fields [1,2,3,4]. Namvar et al. [5] used improved multi-objective particle swarm optimization with a genetic algorithm (MOPSOGA) to study the optimal design of a medium-thickness hexagonal honeycomb sandwich plate—highly accurate optimal velocity, inertia weight, and acceleration coefficient parameters were obtained. Gholami et al. [6] used particle swarm optimization (PSO) technology to optimize the design of a composite sandwich panel with a honeycomb core structure. They found that the optimal geometry of a honeycomb cell had the properties of radius and thickness converging to their bottom bounds, while its length converged to its top bound. Martinez-Martin et al. [7] proposed a multi-objective optimization method to design the material properties of honeycomb sandwich panels with minimum weight and maximum thermal resistance. The program provided a simplified tool to quickly shrink lightweight, insulated sandwich panel structures. Based on the design of 28 finite element simulation samples, Lim et al. [8] used the Kriging method to establish an alternative model for the performance optimization design of the hybrid sandwich panel (HSP). The HSP was optimized to achieve two conflicting goals: light weight and high energy absorption. The total panel thickness was limited to 100 mm. Over the past few decades, researchers have conducted in-depth studies into the low-velocity impact of sandwich structures [9,10]. Liu et al. [11] studied the influence of impactor shape on low-velocity impact behavior by combining experimental and numerical simulation methods. They found that the damage—consisting of fiber damage, matrix damage, panel delamination, and core member buckling—was dependent on the impactor shape, impact energy, and impact location. Qin et al. [12] carried out an experimental study on a fully clamped sandwich plate impacted by a hemispherical head hammer at low speed. They found that the impact position had an important influence on the dynamic response of the sandwich plate under low-velocity impact. The impact resistance of the sandwich plates decreased gradually from the central position to the non-central position. 

In recent years, there have also been breakthroughs in high-speed, hypervelocity, and explosion impacts [13]. Zhang et al. [14] established partial differential equations of a honeycomb sandwich plate based on Reddy’s higher shear deformation theory and Hamilton’s principle. The effects of geometric parameters and explosive loads on the plate were obtained. Pydah et al. [15] analyzed the transient elastoplastic deformation of a double-core sandwich panel under explosive load. The study found that for a given blast load, compared with equal-weight wall panels without a protective cover, the use of a protective cover could significantly reduce the energy dissipation of the sandwich panel, the maximum centroid deflection of the bottom panel, and the maximum plastic strain of the core. Theobald [16] compared the anti-explosion performance of foamed aluminum and honeycomb sandwich panels under an air-impact load. They found that Alporas and honeycomb cores could provide higher relative performance with thicker face sheets. Under the majority of the loading conditions investigated, the thick core honeycomb panels show the greatest increase in blast resistance of the core materials. At the same time, excellent progress has been made in the study of vibration and the vibration reduction of sandwich structures [17,18,19]. In addition, other research work has made significant breakthroughs, including work on the vibration characteristics of the magnetorheological sandwich in different magnetic fields, 3D scanning technology to evaluate the surface damage of honeycomb panels, and the flexible automatic production of sandwich panels [20,21].

In order to improve the protection ability of traditional honeycomb structure, a compound double layer honeycomb structure was designed. The military material 2024 aluminum and 4340 steel are applied to obtain better protection performance. At the same time, a calculation method which can reflect the deformation characteristics of the composite double layer honeycomb structure is proposed. Compared with the energy absorption rate, this method can effectively distinguish the variation trend of similar structures at different times. The best protection structure scheme is obtained.

This paper will elaborate the research content through four sections: in Section 1, this paper introduces of research status of honeycomb structure. In Section 2, based on the current research trend, a composite double layer honeycomb structure is designed and the fracture energy calculation method of the composite double layer honeycomb structure is applied to the model. In Section 3, the mechanical properties of the structure are analyzed by numerical simulation. Section 4 summarizes the research content of the paper.

## 2. Structural Design and Material Model

### 2.1. Structural Design

**Figure 1:**
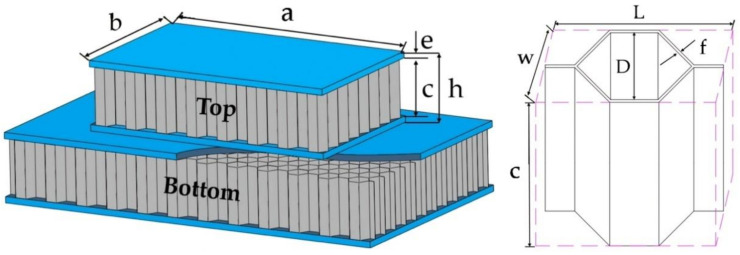
Structure of honeycomb sandwich.

In this study, the cells of the composite double-layer honeycomb structure were hexagonal (six sides equal). The honeycomb structure and cells are shown in Figure 1. The honeycomb structure is composed of the core, upper skin, and lower skin bonded together. The impact bar was a cylinder. The impact bar is approximated as a rigid body. Its diameter was 16 mm, and its length was 25 mm.

Where Top represents the upper honeycomb structure, and Bottom represents the lower honeycomb structure. The structure size of the honeycomb sandwich is shown in Table 1. The thickness of skin, honeycomb core, and cell wall are constant in size. f, w, and l are variables.

In this study, a total of 8 kinds of structures were designed to analyze the effects of different structure sizes and material on the mechanical properties, as shown in Table 2.

Where Al/St indicates that the top core material is 2024 aluminum (Xingyuan Hardware, Taiyuan, China.) and the bottom core material is 4340 steel (Yongchuang Metal products Co. LTD, Dongguan, China.), St/Al means the top core material is 4340 steel, the bottom core material is 2024 aluminum.

### 2.2. Material Model

When the velocity is 50–1000 m/s in the collision process, the material is mainly plastic and viscous deformation [22]. During the deformation process of the honeycomb structure, the honeycomb cells at the edge of the impact area deform in the radial direction under tension, and finally break, which is a typical tensile tear type failure. Tensile tear failure is caused by tensile tear dimple, which is one of the three typical types of micropore fracture mechanism. Studies show that the skin and core of honeycomb sandwich structure are formed by bonding, which results in separation and sliding of contact surfaces during high-speed impact. Therefore, to approximate the actual situation, the contact between the skin and core is a fixed connection failure method. The contact surface of the skin and core is firmly connected, allowing the two surfaces to slide and separate each other after failure. The deformation of the honeycomb core is a complex process. In addition to the contact between the skin and the core, the honeycomb cells themselves also interact with each other. Therefore, the honeycomb core is set automatically in face to face contact. At present, the common grid construction software includes ICEM, HyperMesh, Truegrid, etc. [23]. The preprocessing of LS-DYNA solver can be carried out in various ways. In this paper, Truegrid software 3.1.3 is used for modeling, and the honeycomb structure adopts a Lagrangian grid, all of which are hexahedral solid elements with eight notes. At the same time, the pretreatment of finite element model is set up by k file. In order to ensure the calculation accuracy, the grid of the honeycomb structure in the impact area is denser. The time step of calculation is reduced, so as to increase the number of iterations.

Composite double-layer honeycomb structure adopts Lagrangian algorithm, material models are selected JOHNSON_COOK model and GRUNEISEN equation of state. The flow stress of the material is expressed as the product of the strain function, strain rate function, and temperature function. It can reflect the effects of strain hardening, strain rate strengthening, and temperature softening. Its expression is

(1) $$\sigma =\left(A+B\varepsilon^{pn}\right)\left(1+Cln{\dot{\varepsilon }}^{*}\right)\left(1-T^{*m}\right)$$

where *σ* is the plastic flow stress of the material; *ε^p^* is the equivalent plastic strain; $\dot{\varepsilon^{*}}$ is the relative equality plastic strain rate; T^*^ is the dimensionless temperature; *A* is the initial yield stress at the reference temperature and the reference strain rate, *B* is the strain hardening modulus of the material, *C* is the strain rate sensitivity coefficient, *n* is the hardening exponent of the material, *m* is the temperature softening coefficient.

2024 aluminum and 4340 steel were selected as the honeycomb core materials in the composite double-layer honeycomb sandwich structure. The specific parameters of the material model are listed in Table 3.

**Figure 2:**
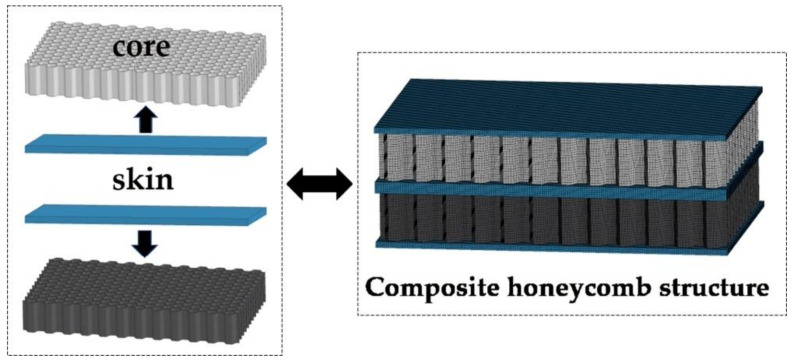
Finite element model of composite honeycomb structure.

The composite honeycomb structure adopts hexahedral solid element with eight nodes. Because the composite model is symmetrical, to reduce the number of calculation units, a quarter model is established for calculation. Due to different structural dimensions, there are 302,904 solid elements for 1# and 5#, 245,556 solid elements for 2#, 3#, 6# and 7#, and 217,512 solid elements for 4# and 8#. The displacement of the edge of the composite honeycomb structure is prevented, so the freedom of the solid elements of the edge in the X, Y, and Z directions is limited. The remaining solid elements are all six degrees of freedom, with respect to displacement and rotation of X, Y, and Z. The composite honeycomb structure is formed by bonding two honeycomb sandwich plates of different materials. The finite element model is shown in Figure 2.

### 2.3. Calculation of Fracture Energy of Composite Honeycomb Structure

At present, the energy absorption rate is the main index to measure the mechanical properties of honeycomb structures. With the introduction of the concept of fracture energy, the fracture characteristics of materials are well expressed. Further study on the honeycomb structure found that the energy absorption characteristics of the honeycomb core under high-speed impact are mainly manifested in three aspects: the energy absorbed by the plastic deformation of the honeycomb cell under the impact rod, energy absorbed by friction between impact rod and honeycomb core, and the energy absorbed by the tearing of the honeycomb cells around the impact rod after high-speed impact—this part of energy is called ‘fracture energy’. The semi-empirical expression for the fracture of composite honeycomb structures is presented below.

The total energy absorbed by the composite honeycomb structure [24]

(2) $$w=w_{F}+w_{P}+w_{m}$$

where *W* is the total energy absorbed by the honeycomb, *W_F_* is the energy absorbed by the fracture of the honeycomb cell, *W_P_* is the energy absorbed by the plastic deformation of the honeycomb, *W_m_* is the energy absorbed by the honeycomb friction.

(3) $$w=\int_{0}^{t}f_{\left(t\right)}dt$$

(4) $$w_{p}=\int_{0}^{t}f_{P}\left(t\right)dt$$

(5) $$w_{m}=\int_{0}^{t}f_{m}\left(t\right)dt$$

where, *f*_(*t*)_ is the energy absorption curve of honeycomb per unit time, *f_P_*(*t*) is the plastic deformation energy absorption curve of the honeycomb, *f_m_*(*t*) Is the friction energy absorption curve of the honeycomb.

(6) $$w_{F}=\int_{0}^{t}f_{\left(t\right)}dt-\int_{0}^{t}f_{P}\left(t\right)dt-\int_{0}^{t}f_{m}\left(t\right)dt$$

Then the approximate expression of fracture energy is

(7) $$FG=\frac{w_{F}}{A}$$

*A* is the area of the fracture surface perpendicular to the tensile stress direction, and its expression is

(8) $$A=f\cdot M\left(\int_{0}^{t}\nu_{\left(t\right)}dt-ne\right)$$

*M* is the number of cells covered by the impact rod. *V*_(*t*)_ is the residual speed curve. It is a function of time. *f* is the cell wall thickness, *n* is the number of skins, *e* is the skin thickness. For composite structures with different cell diameters (when D = 3 mm M = 18, D = 6 mm M = 12)

(9) $$M\left(\int_{0}^{t}\nu_{\left(t\right)}dt-ne\right)=M_{1}\left(\int_{0}^{t}\nu_{\left(t\right)}dt-n_{1}e\right)+M_{2}\left(\int_{0}^{t}\nu_{\left(t\right)}dt-n_{2}e\right)$$

Then the expression of fracture energy is

(10) $$FG=\frac{\int_{0}^{t}f_{\left(t\right)}dt-\int_{0}^{t}f_{P}\left(t\right)dt-\int_{0}^{t}f_{m}\left(t\right)dt_{}}{f\cdot M_{1}\left(\int_{0}^{t}\nu_{\left(t\right)}dt-n_{1}e\right)+f\cdot M_{2}\left(\int_{0}^{t}\nu_{\left(t\right)}dt-n_{2}e\right)}$$

The fracture energy formula is introduced into the model operation and the results are discussed.

## 3. Impact Resistance Analysis of Composite Double-Layer Honeycomb Structure

**Figure 3:**
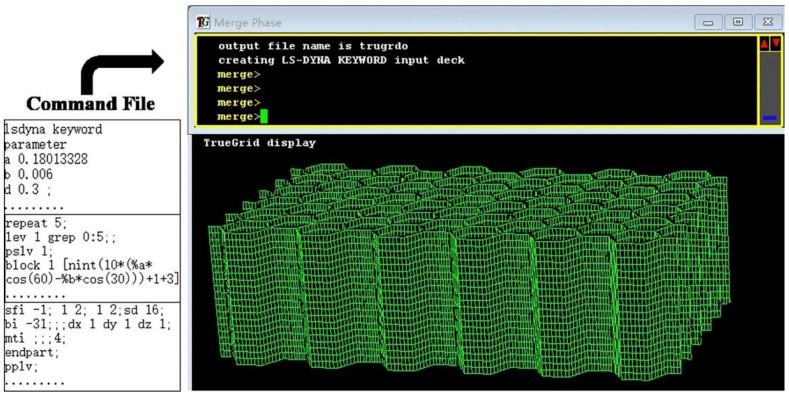
Modeling schematic diagram of honeycomb core.

In this study, the solver uses ANSYS LS-DYNA18.2. The model is built by the TrueGrid software. Due to the different size of the composite honeycomb structure model to be built, its flexibility is required to be high. Therefore, the parametric modeling method is adopted in this study. Model building, mesh encryption, and control constraints by adding a command in the TrueGrid. Take the modeling of honeycomb core as an example, as shown in Figure 3. At the same time, the operation k file of the model is written, and the TSSFAC keyword in *CONTROL_TIMESTEP is reduced, and the time step is shortened, so as to improve the accuracy of the operation results.

**Figure 4:**
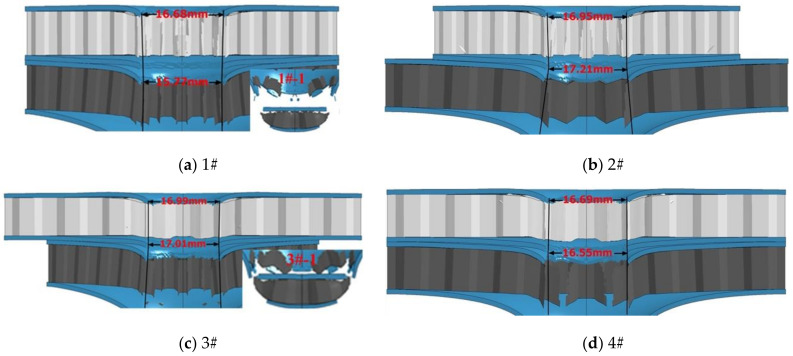
Damage results of 1#–4# structure. (**a**) Damage result of 1# structure; (**b**) Damage result of 2# structure; (**c**) Damage result of 3# structure; (**d**) Damage result of 4# structure.

In order to analyze the energy absorption characteristics and failure forms of the composite honeycomb structure, a numerical simulation analysis was carried out on a model composed of different structure sizes and materials. The damage results of the structure are shown in Figure 4.

In Figure 4, the diameter of the holes in the bottom layer of the four kinds of structures are 17.68, 18.47, 17.25, and 15.70 mm, respectively. The top layer is 2024 aluminum and the bottom layer is 4340 steel. During the impact process, the bottom layer 1# and 3# with smaller cell diameter fracture process produces large fragments (1#-1 and 3#-1).

**Figure 5:**
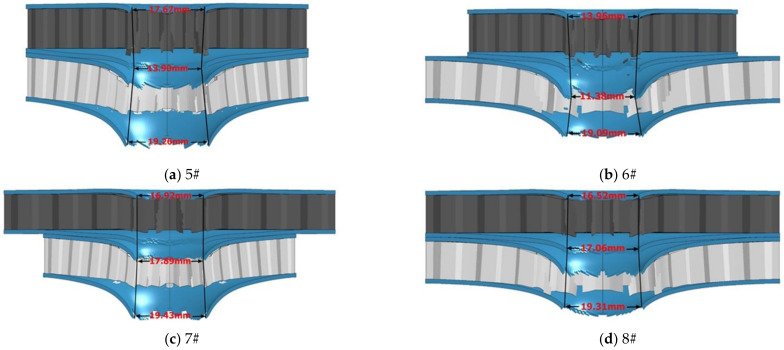
Damage results of 5#–8# structure. (**a**) Damage result of 5# structure; (**b**) Damage result of 6# structure; (**c**) Damage result of 7# structure; (**d**) Damage result of 8# structure.

**Figure 6:**
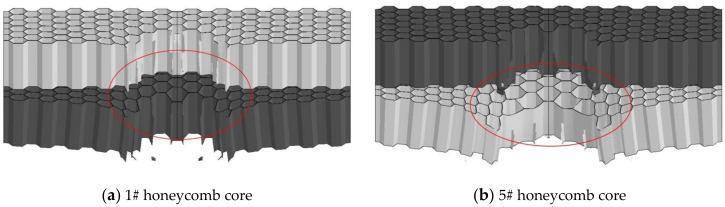
Damage results of 1# and 5# honeycomb core. (**a**) Damage result of 1# honeycomb core; (**b**) Damage result of 5# honeycomb core.

For the 5#–8# structure (Figure 5) with the top core of 4340 steel and the bottom core of aluminum core, the diameter of the center hole is respectively 13.90, 11.38, 17.89, and 17.06 mm. Due to the good ductility of skin material, the underlying skin deformation is too large. The shape of the opening is similar to the hourglass shape, and the diameter of the opening of the middle skin is related to the tensile deformation of the underlying material. The tensile deformation of the bottom cell is too large, resulting in the reduction of tear aperture, as shown in Figure 6.

It can be seen in Figure 6b that the honeycomb cells in the impact region of the structure with aluminum in the bottom layer are severely deformed under tensile stress, and the material is close to the axis, leading to smaller hole diameter. The diameter of the center hole is 15.77 mm and 13.90 mm, respectively. Due to excessive deformation, the top and bottom cores are separated seriously.

**Figure 7:**
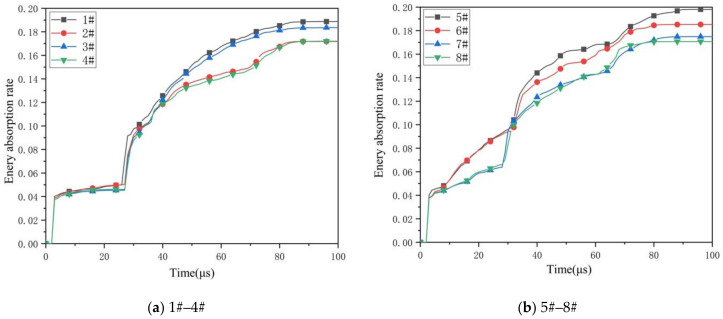
Energy absorption rate curves. (**a**) Energy absorption rate curves of 1#–4#; (**b**) Energy absorption rate curves of 5#–8#.

Figure 7 shows the energy absorption rate curves of 8 kinds of composite honeycomb structures. The energy absorption rate [25] is an indirect expression of the energy absorption characteristics of the honeycomb structure through the velocity. The curve appears at a point of intersection after 30 μs. At this time, the remaining speed is the same. At the moment when the impact rod and the second layer of honeycomb sandwich panel just contact, it can be seen that the remaining speed is greatly affected by the skin. Since the energy absorption rate is an indirect representation method, this also leads to the inability to reflect the change of the micro-element in each time period. Especially in the case of the same material combination, the composite honeycomb structure with the same top cell diameter, no matter how the size of the bottom structure changes, the two curves are about to overlap, and the difference between the structures cannot be effectively distinguished. In order to analyze the influence of structural characteristics on mechanical properties at each moment, the fracture energy calculation method of composite honeycomb structure proposed in this paper is applied. The following figure shows the curve of fracture energy of 1# structure with time.

**Figure 8:**
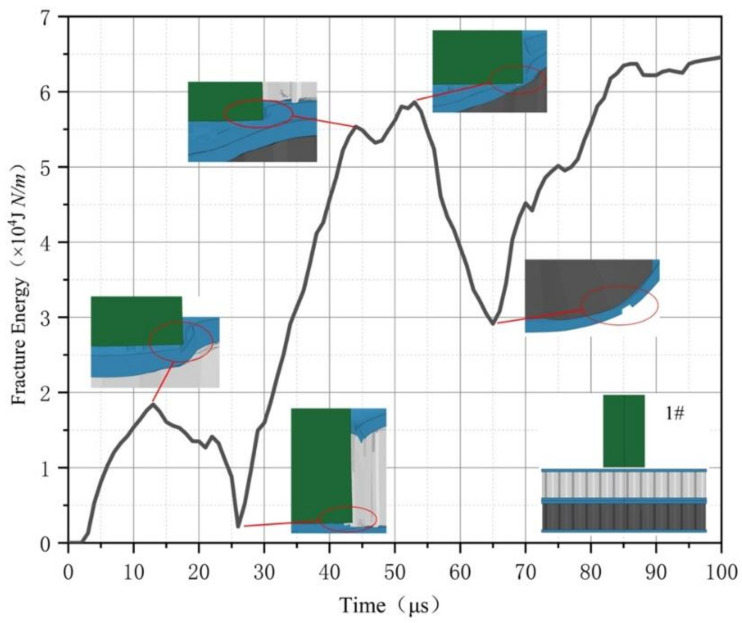
Curve of 1# fracture energy.

**Figure 9:**
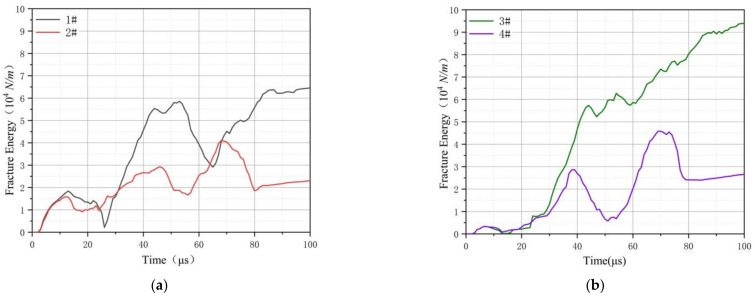

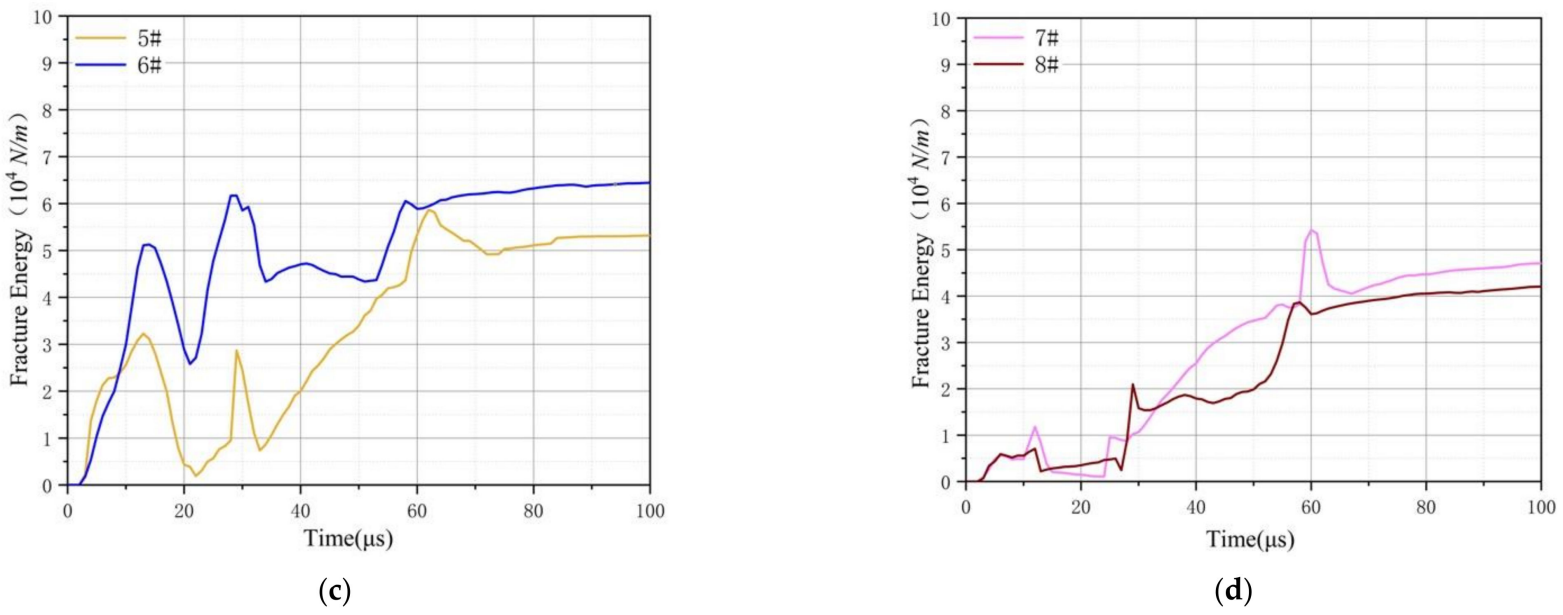
Fracture energy curves of 1#–8# composite structures. (**a**) Fracture energy curves of 1# and 2# composite structures; (**b**) Fracture energy curves of 3# and 4# composite structures; (**c**) Fracture energy curves of 5# and 6# composite structures; (**d**) Fracture energy curves of 7# and 8# composite structures.

The calculation time of the composite structure is set to 100 μs. It can be seen from Figure 8 that the composite honeycomb structure increases with plastic deformation. The top skin reaches the crack initiation point first at about 12 μs, and the plastic deformation of the top skin reaches the maximum. At this time, the material began to lose stability and fracture, and the curve began to drop. At about 25 μs, the aluminum core is completely destroyed, and the fracture energy reaches a minimum. Because the strength of the underlying material is large, and this structure is composed of two honeycomb sandwich structures. As a result, the thickness of the middle skin is twice that of the edge skin, so the fracture energy increases sharply after 25 μs, and the deformation of the bottom honeycomb panel gradually increases. The bottom skin of the top aluminum core honeycomb sandwich structure began to fracture at about 42 μs, and the top skin of the bottom steel core honeycomb sandwich structure began to fracture at about 53 μs. Both skins produced small peaks. At about 65 μs, the bottom honeycomb structure began to be destroyed, and energy was accumulated for the generation of impact fragments, which caused the fracture energy curve to rise until the structure was completely penetrated. Both the fracture energy and the energy absorption rate can show the influence of the skin on the structure, but the fracture energy can also express the change trend and damage of the structure in each time period. Figure 9 is the fracture energy curve of eight kinds of composite structures.

When the impact rod penetrates into the area where the steel is the honeycomb core, the curve fluctuates greatly. In this area, the fluctuation value of every two curves differs by about twice. By comparing with the structure parameters, it is found that the difference of the curve is related to the cell diameter of the structure, and the relationship is linear. The energy absorption of the eight kinds of structures is calculated. Energy per mass and energy per volume of the structures are shown in Table 4.

It can be seen from Table 4 that the maximum of the energy per mass and energy per volume is 5# structure, 53.567 J/g and 167.867 J/cm^3^. The minimum is 4# structure, 26.462 J/g and 78.920 J/cm^3^. In the case of the same structure size, the structure with the top layer of steel core has a larger energy absorption than the structure with the top layer of aluminum core. It can be seen that the higher the strength of the top layer material, the greater the impact on the overall energy absorption characteristics. At the same time, the energy absorption characteristics are also related to the overall cell density. The 1# and 5# structures with higher cell density have more energy absorption. The 4# and 8# structures, whose cell density is one-half of them. The energy per mass and energy per volume of 4# is 43.2% and 45.9% less than 1#. 8# is 45.7% and 48.3% less than 5#.

## 4. Conclusions

In this study, TrueGrid was used to mesh and set the boundary conditions of the composite honeycomb structure, and Ls-dyna was used to solve the problem. First, numerical simulation of eight kinds (see Table 2) composite honeycomb structure is carried out. The semi-empirical formula for calculating the fracture energy of the composite honeycomb structure is used, the fracture energy curve of the composite structure is obtained. Further study the influence of the composite mode of the honeycomb structure on the mechanical properties of the structure, and the following conclusions are obtained:

(1) It is feasible to use TrueGrid software to divide finite element mesh and densified mesh. The finite-element mesh with high-quality mesh can be obtained. The method of increasing the number of iterations can obtain satisfactory numerical simulation results. The proposed method for calculating the fracture energy of the composite honeycomb sandwich structure can well describe the deformation trend and damage of the structure in each time period, especially for the judgment of the skin fracture time.
(2) Cell density is one of the main factors affecting the fracture energy of composite honeycomb structure. With the increase of cell density, the greater the fracture energy, but the influence of cell density is less than that of material strength.
(3) When the material and structure size of the aluminum core layer are the same, the ratio of the fracture energy in the 4340 steels area is the same as the ratio of the cell diameter in this area.
(4) In the eight kinds of structures, under the same structural size, the structure with 4340 steel on the top layer has a larger energy per mass and energy per volume absorption than the structure with 4340 steel on the bottom layer. The maximum energy per mass and energy per volume are 53.567 J/g and 167.867 J/cm^3^, which are 9.98% and 23.77% higher than the traditional aluminum honeycomb structure.

## Figures and Tables

**Table 1:** Dimensions of the honeycomb sandwich.

D (mm)	a × b (mm^2^)	e (mm)	c (mm)	h (mm)	F (mm)
3	64 × 40	1	9	11	0.06
6	84 × 50	1	9	11	0.06

**Table 2:** Honeycomb material structure dimension match.

Skin	Scheme	Materials	Top	Bottom	Scheme	Materials	Top	Bottom
Aluminum	1#	Al/St	D = 3 mm	D = 3 mm	5#	St/Al	D = 3 mm	D = 3 mm
	2#	Al/St	D = 3 mm	D = 6 mm	6#	St/Al	D = 3 mm	D = 6 mm
	3#	Al/St	D = 6 mm	D = 3 mm	7#	St/Al	D = 6 mm	D = 3 mm
	4#	Al/St	D = 6 mm	D = 6 mm	8#	St/Al	D = 6 mm	D = 6 mm

**Table 3:** Material parameters.

Material	*ρ* (g/cm^3^)	G (GPa)	A (GPa)	B (GPa)	n	C	m	*T_m_* (*K*)	*T_r_* (*K*)
2024 Aluminum	2.785	28.6	0.265	4.26	0.34	0.015	1.00	775	300
4340 Steel	7.83	77	0.792	0.51	0.26	0.014	1.03	1793	300

**Table 4:** Energy per mass and energy per volume.

Scheme	Energy Per Mass (J/g)	Energy Per Volume (J/cm^3^)	Scheme	Energy Per Mass (J/g)	Energy Per Volume (J/cm^3^)
1#	46.596	146.021	5#	53.567	167.867
2#	32.552	98.553	6#	41.368	126.590
3#	37.420	114.509	7#	36.437	110.315
4#	26.462	78.920	8#	29.069	86.778

## Data Availability

The data presented in this study are available on request from the corresponding author. The data are not publicly available due to programming privacy in structural design.
